# *Lrig2*-Deficient Mice Are Protected against *PDGFB*-Induced Glioma

**DOI:** 10.1371/journal.pone.0073635

**Published:** 2013-09-04

**Authors:** Veronica Rondahl, Camilla Holmlund, Terese Karlsson, Baofeng Wang, Mahmood Faraz, Roger Henriksson, Håkan Hedman

**Affiliations:** 1 Department of Radiation Sciences, Oncology, Umeå University, Umeå, Sweden; 2 Regionalt Cancercentrum Stockholm, Karolinska Universitetssjukhuset Solna, Stockholm, Sweden; NIH/NCI, United States of America

## Abstract

**Background:**

The leucine-rich repeats and immunoglobulin-like domains (LRIG) proteins constitute an integral membrane protein family that has three members: LRIG1, LRIG2, and LRIG3. LRIG1 negatively regulates growth factor signaling, but little is known regarding the functions of LRIG2 and LRIG3. In oligodendroglial brain tumors, high expression of LRIG2 correlates with poor patient survival. *Lrig1* and *Lrig3* knockout mice are viable, but there have been no reports on *Lrig2*-deficient mice to date.

**Methodology/Principal Findings:**

*Lrig2*-deficient mice were generated by the ablation of *Lrig2* exon 12 (*Lrig2E12*). The *Lrig2E12-/*- mice showed a transiently reduced growth rate and an increased spontaneous mortality rate; 20-25% of these mice died before 130 days of age, with the majority of the deaths occurring before 50 days. *Ntv-a* transgenic mice with different *Lrig2* genotypes were transduced by intracranial injection with platelet-derived growth factor (PDGF) B-encoding replication-competent avian retrovirus (RCAS)-producing DF-1 cells. All injected *Lrig2E12+/+* mice developed *Lrig2* expressing oligodendroglial brain tumors of lower grade (82%) or glioblastoma-like tumors of higher grade (18%). *Lrig2E12-/*- mice, in contrast, only developed lower grade tumors (77%) or had no detectable tumors (23%). *Lrig2E12-/*- mouse embryonic fibroblasts (MEF) showed altered induction-kinetics of immediate-early genes *Fos* and *Egr2* in response to PDGF-BB stimulation. However, *Lrig2E12-/*- MEFs showed no changes in Pdgfrα or Pdgfrβ levels or in levels of PDGF-BB-induced phosphorylation of Pdgfrα, Pdgfrβ, Akt, or extracellular signal-regulated protein kinases 1 and 2 (ERK1/2). Overexpression of LRIG1, but not of LRIG2, downregulated PDGFRα levels in HEK-293T cells.

**Conclusions:**

The phenotype of *Lrig2E12-/*- mice showed that *Lrig2* was a promoter of PDGFB-induced glioma, and *Lrig2* appeared to have important molecular and developmental functions that were distinct from those of *Lrig1* and *Lrig3*.

## Introduction

Oligodendroglial tumors, including oligodendroglioma, anaplastic oligodendroglioma, oligoastrocytoma, and anaplastic oligosastrocytoma, are diffusively infiltrating primary brain tumors [1]. Although many oligodendroglial tumors respond favorably to therapy, most patients are not cured by current treatment modalities. The most common cytogenetic chromosomal alteration in oligodendroglial tumors is a combined loss of chromosomes 1p and 19q, which is associated with improved survival and response to treatment [2]. Platelet-derived growth factor (PDGF) and the PDGF receptors, PDGFRα and PDGFRβ, are overexpressed in the majority of oligodendrogliomas [3]. A functional role for dysregulated PDGFR signaling in oligodendroglial tumors is further supported by animal models in which PDGF autocrine stimulation contributes to gliomagenesis, including oligodendroglioma genesis [4,5].

Leucine-rich repeats and immunoglobulin-like domains (LRIG) proteins are a family of integral membrane proteins [6–9]. The mammalian LRIG gene family is composed of three paralogues, *LRIG1*, *LRIG2*, and *LRIG3* [9]. The best-studied family member, LRIG1, antagonizes growth factor signaling mediated by the ERBB [10,11], MET [12], and RET [13] receptor tyrosine kinases, and is suggested to be a tumor suppressor [7,14,15]. LRIG1 expression is associated with a favorable prognosis in many cancer types [16–20]. *Lrig1* knockout mice show hyperproliferation of intestinal stem cells [21,22] and spontaneously develop intestinal tumors [22]. Furthermore, Lrig1 regulates epidermal stem cell quiescence [23,24], and the knockout mice develop psoriatic skin lesions, suggesting that Lrig1 also has an essential role in epidermal homeostasis [25]. This role is further supported by the redistribution of LRIG proteins observed in human psoriatic skin lesions compared with the normal epidermis [26]. In the nematode *Caenorhabditis elegans*, the single LRIG paralogue, SMA-10, regulates body size and bone morphogenetic protein signaling [27]. LRIG3 appears to have a role in modulating Fgf and Wnt signaling in *Xenopus* [28]. *Lrig3*-deficient mice have cranio-facial and inner ear defects, but appear grossly normal in other respects [29]. Little is known regarding the molecular and developmental functions of mammalian LRIG2. Recently it was found that *LRIG2* mutations are associated with congenital urofacial syndrome [30]. In addition, LRIG2 expression is an independent prognostic factor associated with poor survival in oligodendroglioma [31] and squamous cell carcinoma of the uterine cervix [32]. The latter suggests that LRIG2 might promote the genesis or growth of oligodendroglial tumors and cervical squamous cell carcinoma, and that LRIG1 and LRIG2 might have different, possibly opposing, functions.

In this study, we generated *Lrig2*-deficient mice and investigated their survival, health, growth, and susceptibility to PDGFB-induced gliomagenesis. In addition, we studied possible effects of Lrig2 on Pdgfr levels and PDGF-induced intracellular signaling events.

## Materials and Methods

### Ethics Statement

All mice were housed and maintained, and all experiments were performed in accordance with the European Communities Council Directive (86/609/EEC). Experimental protocols were approved by the Regional Ethics Committee of Umeå University, Umeå, Sweden (registration nos. A12-07, A5-2010, A25-07, and A52-10).

### Generation of *Lrig2*-Deficient Mice and Genotyping

**Figure 1:**
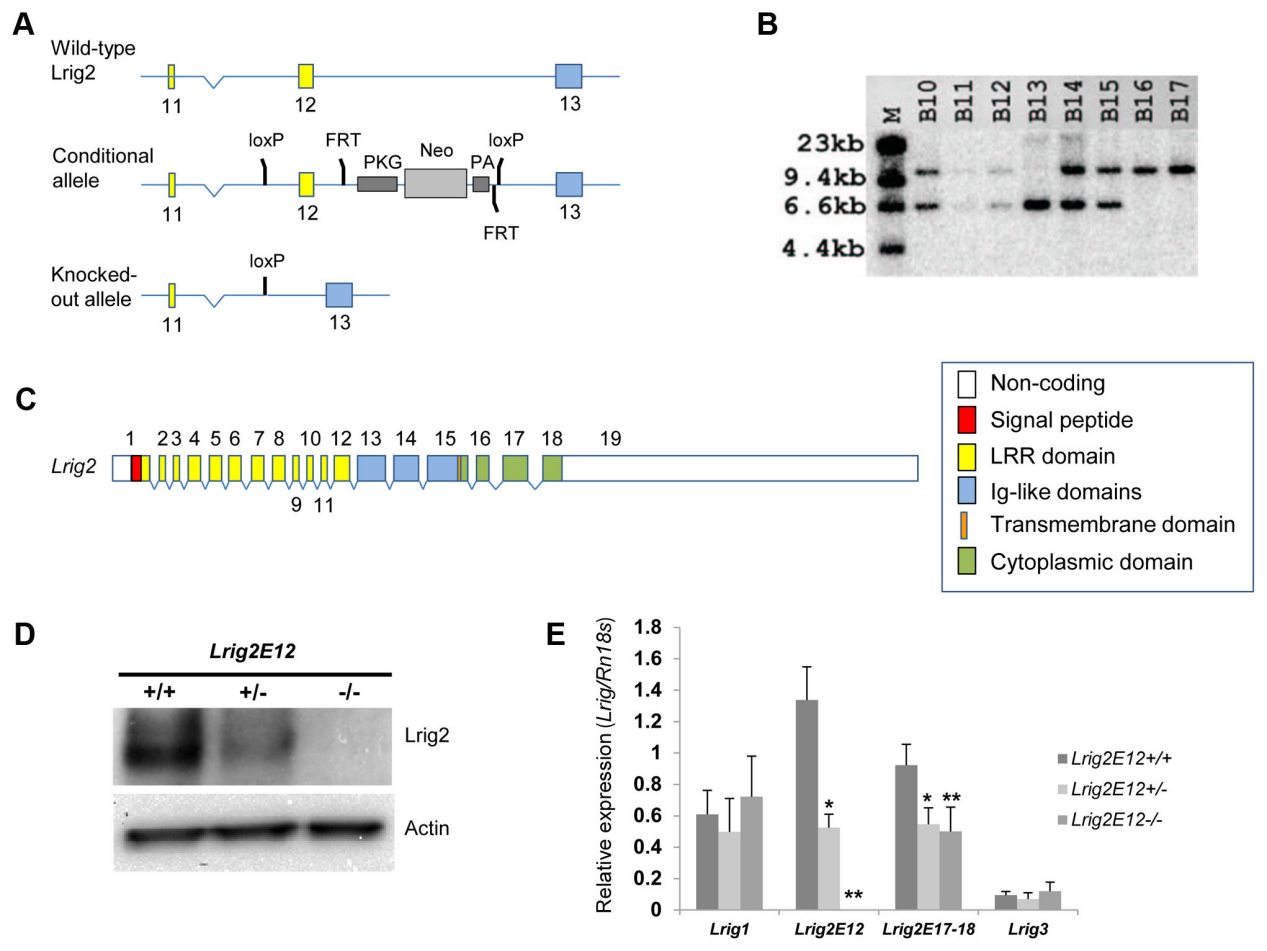
Genomic organization of the mouse *Lrig2* gene and the generation and molecular analyses of *Lrig2-*deficient mice. (**A**) Schematic drawing of the wild-type, conditional, and disrupted *Lrig2* alleles. A PKG-neo selection cassette was inserted downstream of exon 12. Exon 12 and the PKG-neo cassette were flanked by *loxP* sites and were deleted together, in a single step, by mating with OzCre mice. Color coding is as in **C**. (**B**) Southern blot using tail DNA isolated from the offspring of an Lrig2E12+/- × Lrig2E12+/- mating. The expected sizes are as follows: wild-type (*Lrig2E12*+) allele, 11.0 kb; and *Lrig2* exon 12-ablated (*Lrig2E12*-) allele, 6.6 kb. Lane B13 is from *Lrig2E12-/*-, lanes B10-12 and B14-15 are from *Lrig2E12+/-*, and the remaining lanes are from *Lrig2E12+/+* mice. (**C**) Genomic organization of the mouse *Lrig2* gene. Gene structure of *Mus musculus Lrig2* is shown. Gene organization was deduced from the sequence of the mouse genome and the *Lrig2* mRNA (GenBank accession number NM_001025067). Exons are indicated with boxes and are drawn to scale. Exon numbers are indicated with numbers. The *Lrig2* gene is approximately 58 kb, and it is located on mouse chromosome 3 F2. Color coding depicts the encoded protein domains, including a signal peptide (red), a leucine-rich repeats domain (yellow), three immunoglobulin-like domains (blue), a transmembrane domain (orange), and a cytosolic domain (green). (**D**) Western blot of MEF cell lines of different *Lrig2* genotypes. Top, anti-Lrig2 polyclonal; Bottom, anti-actin. (**E**) *Lrig* mRNA levels in mice of different genotypes. The mRNA levels of *Lrig1*, exon 12-containing *Lrig2* (*Lrig2E12*), exon 17-18 boundary-containing *Lrig2* (*Lrig2E17-18*), and *Lrig3* in brains of 3-week old mice were analyzed using quantitative real-time RT-PCR. The *Lrig/Rn18s* ratio was calculated and normalized to the corresponding ratio in reference RNA from Stratagene. Shown are the means of wild-type (n=6), *Lrig2E12+/-* (n=5), and *Lrig2E12-/*- (n=8) mice, with error bars indicating the standard deviations. Significant differences compared with the wild-type mice are indicated with asterisks (*p<0.01 and **p<0.001).

Construction of the targeting vector and generation of mice with floxed or deleted *Lrig*2 exon 12 (*Lrig2E12*) alleles was performed at Ozgene Pty Ltd (Bentley DC, WA, Australia). A schematic drawing of the targeting vector is shown in Figure 1A. The 5´, loxP, and 3´ homology arms were generated by polymerase chain reaction (PCR) amplification from the genomic DNA of 129Sv/J mice. The linearized targeting vectors were electroporated into embryonic stem (ES) cells derived from 129Sv/J mice, and transformed colonies were selected using G418. Integration of the targeting vectors by homologous recombination and a lack of random integration were verified through Southern blot analysis (data not shown). Chimeric mice were generated through the injection of ES cells into C57BL/6 blastocysts, and germ-line transmission of the floxed *Lrig2* allele was confirmed through Southern blot analysis (data not shown). To generate *Lrig2*-deficient mice, the *Lrig2E12flox* mice were crossed with Oz-Cre transgenic mice (Ozgene). Genomic deletion of *Lrig2E12* was confirmed through Southern blot analysis (Figure 1B). The *Cre* gene was removed by back-crossing the mice against C57BL/6 and confirmed through Southern blot analysis (data not shown). For genotyping, tail DNA was extracted using the REDExtract-N-AMP Tissue PCR kit (Sigma-Aldrich Sweden AB, Stockholm, Sweden) and amplified using PCR with the reaction mix included in the kit. The following three primers were used: 5´ *Lrig2E12*-ablated, TGGGAGTGAGCTAGGCAG; 5´ *Lrig2*-wild-type, TGCACTAGGCAGTCTTAAACCA; and 3´ *Lrig2*, TCAGGCAGTGACAGAAGGTGTG. The PCR conditions were 95°C for 3 minutes followed by 30 cycles of 95°C for 30 seconds, 64°C for 1 minute, and 72°C for 1 minute, with a final step of 72°C for 10 minutes. The PCR products were separated and visualized using analytical agarose gel electrophoresis.

### cDNA Sequencing

Total RNA was isolated from brain tissue samples from two wild-type and eight *Lrig2E12-/-* mice using the RNeasy Lipid Tissue kit (Qiagen AB, Sollentuna, Sweden) according to the manufacturer’s instructions. cDNA was synthesized from the isolated RNA by reverse transcription and, thereafter, amplified by *Lrig2*-specific PCR. The PCR products were cloned into the pCR-Blunt II-TOPO vector (Life Technologies, Europe BV, Stockholm, Sweden). Nucleotide sequencing was performed using Big Dye Terminator v 3.1 sequencing kit (Applied Biosystems, Stockholm, Sweden) and a 3730x1 DNA analyzer (Applied Biosystems).

### Tissue and Cell Lysis, SDS PAGE, and Immunoblotting

Embryos and cells used for the determination of starvation-induced Pdgfr expression levels were lysed in lysis buffer (1% Triton X-100, 50 mM Tris-HCl, pH 7.5 and 150 mM NaCl) supplemented with complete protease inhibitor cocktail (Roche Diagnostics, Scandinavia AB, Bromma, Sweden). To determine the levels of phosphorylated Akt and extracellular signal-regulated protein kinases 1 and 2 (Erk1/2), serum-starved cells were lysed in ice-cold lysis buffer supplemented with 10 mM NaF, 1 mM EGTA, 1 mM NaVP_4_, 20 mM beta-glycerophosphate, and EDTA-free protease inhibitor cocktail (Roche Diagnostics). Protein concentrations were determined using a BCA protein assay (Pierce Biotechnology Inc, Rockford, IL, USA), and equal amounts of proteins were separated by sodium-dodecyl-sulfate (SDS) poly-acryl-amide gel electrophoresis (PAGE) and analyzed through Western blotting. Detection of bound primary antibodies was performed using horseradish peroxidase-conjugated secondary antibodies. Antibody-specific protein bands were visualized by using ECL advanced reagent (GE Healthcare, Uppsala, Sweden) and quantified using the Chemidoc XRS system and Quantity One software (Bio-Rad Laboratories, Hercules, CA, USA).

### Antibodies

The polyclonal antibody mLrig2-147 against the synthetic peptide CHERMTENLPFSQRS (single letter amino acid code) in the cytosolic tail of mouse Lrig2 was raised in a rabbit. The resulting antiserum was affinity purified as previously described [33]. The antibody was produced in collaboration with AgriSera (Vännäs, Sweden). The rabbit anti-LRIG1 antibody LRIG1-151 has previously been described [33]. The following primary antibodies from the respective suppliers were also used: rabbit-anti-PDGFRα (#3164), rabbit-anti-PDGFRβ (#3169), mouse-anti-phospho-tyrosine (#9411), rabbit-anti-p44/42 (#9102), rabbit-anti-Akt (#9272), rabbit-anti-phospho-Akt (#9271), and mouse-anti-phospho-p44/42 (#9106) from Cell Signaling (Danvers, MA, USA); rat-anti-PDGFRα (ab90967), rat-anti-PDGFRβ (ab91066), and mouse-anti-Actin (Ab3280) from Abcam (Cambridge, UK); rabbit-anti-PDGFRα (c-20, sc-338) and rabbit-anti-PDGFRβ (p-20, sc-339) from Santa Cruz Biotechnology, Inc. (Santa Cruz, CA, USA); rat-anti-HA (11867423001) from Roche Diagnostics; mouse-anti-acetylated-tubulin (T6793) and mouse-anti-FLAG M2 (F3165) from Sigma-Aldrich; and monoclonal anti-actin (MAB1501) from Millipore AB (Solna, Sweden). Horseradish peroxidase-conjugated secondary antibodies were purchased from GE Healthcare. Species-appropriate Alexa Fluor^488^, Alexa Fluor^555^, or Alexa Fluor^568^-conjugated secondary antibodies were purchased from Molecular Probes (Life Technologies).

### RNA Extraction and Quantitative Real-Time RT-PCR

RNA was prepared from tissues or cells using RNAqueos RNA (Life Technologies) followed by digestion of contaminating DNA using TURBO DNA-free kit (Life Technologies) according to the manufacturer’s instructions. Quantitative reverse transcriptase (RT)-PCR was performed as previously described [9]. Triplicate samples of 20 ng total RNA were analyzed using qScript 1-Step qRT-PCR Kit (Quanta Biosciences, Gaithersburg, MD, USA) according to manufacturer’s instructions on a CFX96 Real-Time System C1000 Thermal Cycler (Bio-Rad Laboratories). The following TaqMan gene expression assays were purchased from Applied Biosystems: *Lrig1* (Mm00456116_m1), *Lrig2E17-18* (Mm01305504_m1), *Fos* (Mm00487425_m1), and *Egr2* (Mm00456650_m1). The following primer and probe sets have been described previously: *Lrig2E12* [9], *Lrig3* [9], and *Rn18s* (also called *18S rRNA*) [7]. Relative gene expression was calculated for each gene using standard curves that were generated using total RNA from an EGF stimulated MEF cell line. All mRNA values were normalized against the corresponding *Rn18s* level in the respective sample. The results were expressed as the specific mRNA/*Rn18s* ratio on an arbitrary scale. For analysis of *Lrig* levels in the brain, the specific mRNA/*Rn18s* ratios were further normalized to the corresponding ratio in QPCR Mouse Reference Total RNA (Agilent Technologies, Santa Clara, CA, USA).

### Survival Analysis

Survival of the mice was recorded by categorizing animals according to cause of death: found dead in cage, euthanized sick (i.e., due to disease symptoms); or euthanized healthy (i.e., due to either use in an experiment or were not needed). These records were then analyzed using Kaplan-Meier plots. Approximately one-third of the mice that were found dead or euthanized due to symptoms of illness were dissected, and their organs were inspected for macroscopic signs of disease. In addition, five *Lrig2E12-/-* mice that were euthanized due to illness symptoms were submitted for necropsy at the National Veterinary Institute (SVA), Uppsala, Sweden. The mice were euthanized at the animal facility by exposure to 80% CO_2_ and 20% O_2_. The abdomen and thorax were opened, and the entire animal was fixed in phosphate-buffered 4% formaldehyde for at least 24 h. The fixed bodies were stored in 70% ethanol until submission to the SVA. At the SVA, the tissue samples were collected, dehydrated, embedded in paraffin, sectioned, stained with hematoxylin-eosin and analyzed.

### Body Weight Records

At 3 weeks of age, the pups were weaned and individually marked with ear notches, and their sex was determined. In addition, their tail tips were collected for genotyping. Growth curves were determined by weighing the mice once a week from 3 until 10 weeks of age. The body weights of embryos (E13.5), 0-day-old pups, 5-day-old pups, 12-week-old adult mice, and 15 to 18-week-old adult mice were recorded. In each age-group, at least eight animals were analyzed per genotype. For embryo collection, virgin females were mated, and 13 days after plug observation, they were euthanized by cervical dislocation. The uterus was immediately removed and placed in cold DMEM. The embryos were then dissected and weighed, and the amnion was used for genotyping. The 0-day-old and 5-day-old pups were weighed and then euthanized by decapitation, and their tail tips were collected for genotyping.

### Anatomical and Histological Analyses of Healthy Mice

Organs were collected from healthy 12-week-old mice. Surgical anesthesia was induced by intraperitoneal injection of a mixture of ketamine (100 mg/kg; Ketaminol® vet, Intervet) and xylazine (10 mg/kg; Narcoxyl® vet, Intervet). The liver, kidneys, spleen, lungs, heart, brain, prostate, seminal vesicles, ovaries, and ovarian tubes were collected, weighed and fixed in phosphate-buffered 4% formaldehyde for 24 hours and then stored in 70% ethanol until further processing. For routine histological analyses, the tissues were dehydrated, embedded in paraffin, sectioned and stained with hematoxylin-eosin. The brain tissues were also stained with Luxol blue to visualize myelin.

### Retroviral Transduction of Mice

*Ntv-a* transgenic mice that express the Tv-a avian retrovirus receptor under the control of the *Nestin* promoter [5] were a kind gift from Lene Uhrbom (Uppsala University, Sweden) and Eric Holland (Memorial Sloan-Kettering Cancer Center, NY, USA). The *Ntv-a* transgenic mice obtained were of the FVB/N genetic background [34]. C57BL/6 Lrig2E12+/- mice were mated with *Ntv-a* transgenic mice and the progeny was then backcrossed for one generation with *Ntv-a* mice to generate *Ntv-a+/+; Lrig2E12+/-* mice. These mice were then intercrossed to generate *Ntv-a+/+* mice with the *Lrig2* genotypes *Lrig2E12+/+*, *Lrig2E12+/-*, or *Lrig2E12-/-*. Newborn mice were intracranially injected in the right frontal region, using a 10-µl Hamilton syringe, with 2 µl (2 x 10^5^cells) of RCAS-PDGFB-HA producing DF-1 chicken fibroblasts, as previously described [5]. The mice were checked every day and euthanized due to symptoms of disease or at 12 weeks of age. The mouse brains were removed and fixed in 4% formalin, paraffin embedded, sectioned, stained with hematoxylin-eosin, and analyzed for tumors by a neuropathologist who was blind to the mouse genotypes.

### In Situ Hybridization

*In situ* hybridization for *Lrig2* RNA transcripts was performed using RNAscope (Advanced Cell Diagnostics, Inc., Hayward, CA, USA) according to the manufacturer’s instructions. An *Lrig2*-specific probe set (Probe -Mm-Lrig2, 310531) was used for staining of tissue sections from formalin-fixed and paraffin-embedded tumor specimens from RCAS-PDGFB-HA infected *Ntv-a; Lrig2E12+/+* mice. As negative control, the RNAscope dapB (310043) control probe set was used. The images were captured with the Pannoramic 250 Flash scanner using a CIS_VCC_F52U25CL camera and a 1.0X distance ring (3DHISTECH Ltd, Budapest, Hungary).

### Cell culture

All cells were cultured at 37°C in a humidified atmosphere containing 5% CO_2_. Primary MEFs were isolated from individual E12.5 or E13.5 embryos obtained from inter crosses of *Lrig2E12+/-* mice on a C57BL/6 genetic background. The fetuses were dissected from the uterus and dechorionated, and the amnion or head was kept for genotyping. The head and the liver were carefully removed, and the remaining embryos were washed in phosphate buffered saline (PBS) and transferred to new dishes. The remaining tissue was minced and trypsinized for 10 minutes at 37°C followed by mechanical disruption using vigorous pipetting. MEFs were cultured in Dulbecco’s modified Eagle’s medium (DMEM) containing 10% fetal bovine serum (FBS) supplemented with 50 µg/ml gentamicin, MEM non-essential amino acids (Life Technologies), and 50 µM 2-mercaptoethanol. The cells were split when at confluence, and kept for up to 6 passages or 14 days. Primary neural cells were established from neonatal mouse brains from *Ntv-a* transgenic mice as previously described [5]. The neural cells were plated on dishes coated with polyornithine and fibronectin in DMEM: Nutrient Mixture F-12 supplemented with N2 supplement, 100 units/ml penicillin, and 100 µg/ml streptomycin (all from Life Technologies) with a daily addition of 10 ng/ml bFGF (PeproTech Nordic, Stockholm, Sweden). *In vitro* infection of the cells was initiated after 3-4 days in culture by adding conditioned media from DF-1 chicken fibroblast cells producing RCAS-PDGFB-HA. After repetitive infection for five days, the cells were seeded on coverslips for immunofluorescence analysis. HEK-293T cells were obtained from Clontech Laboratories Inc. (Mountain View, CA, USA), and cultured in DMEM supplemented with 10% FBS and 50 µg/ml gentamicin.

### Immunofluorescence Microscopy

The cells were grown on glass coverslips overnight to either 70-80% (non-arrested, interphase cells) or 90% confluence, followed by starvation in serum-free medium at the times indicated to induce growth arrest. To determine the levels of activated PDGF receptors, the cells were treated with or without 50 ng/ml PDGF-BB (PeproTech Nordic) for 10 minutes at 37°C. Fixation was performed in 4% paraformaldehyde for 10 minutes at 37°C. The cells were washed three times with 0.1 M glycine in PBS and two times with PBS. In some experiments, the cells were permeabilized and blocked in either PBS containing 5% FBS and 0.1% Triton X-100 (primary cilia length), PBS containing 2% bovine serum albumin and 0.02% saponin (Pdgfr levels), or PBS containing 5% FBS and 0.05% saponin to measure the transduction efficiency of *Ntv-a* transgenic cells for one hour in room temperature. Primary antibodies or isotype-matched control antibodies were diluted in blocking solution and incubated at room temperature for one hour. After washing in PBS, the cells were incubated with fluorescent and species-appropriate secondary antibodies. The cells were washed three times with PBS followed by a final wash in water and mounted onto slides using Prolong Gold (Life Technologies) with DAPI (4, 6-diamidino-2-phenyllindole) for nuclei counterstaining. The images were acquired on a Zeiss LSM 710 confocal microscope and analyzed with Axio Vision (Carl Zeiss AB, Stockholm, Sweden) or IMARIS (Bitplane AG, Zürich, Switzerland) software. For image quantification, the fields of view were selected exclusively on the basis of DAPI fluorescence. Quantification was performed on at least 100 cells for each experiment. Each image represents maximum-intensity projections of acquired z-stacks.

### The *In Situ* Proximity Ligation Assay

Primary MEFs were grown on glass coverslips overnight to 90% confluency followed by starvation in serum-free medium for 24 hours, and stimulation with or without 50 ng/ml PDGF-BB (PeproTech Nordic) for 10 minutes at 37°C. The cells were fixed and permeabilized with ice cold methanol for 10 minutes at -20°C. After washing 3 times with PBS, the *in situ* Proximity ligation assay (PLA) was performed according to the manufacturer’s protocol (Olink Biosciences, Uppsala, Sweden) using mouse-anti-phospho-tyrosine and rabbit-anti-PDGFRα or rabbit-anti-PDGFRβ as primary antibodies.

### Plasmids and Cell Transfections

The expression vector encoding myc-tagged LRIG1 [11] was kindly provided by Colleen Sweeney (UC, Davis, Sacramento, CA, USA); *pcDNA 3.1* was obtained from Life Technologies; and *pcDNA3-PDGFRα* was a kind gift of Carl-Henrik Heldin, (Ludwig Institute, Uppsala, Sweden). The plasmid *p3XFLAG-LRIG2*, encoding FLAG-tagged LRIG2, was generated by PCR-amplification of an *LRIG2* cDNA (RG212836) obtained from OriGene Technologies, Inc. (Rockville, MD, USA) using the primers 5´-TGCAGTTGCTAAGCTTGGTCTCTGCCCCGCGCC-3´ and 5´-TCGACTGGTACCGATATCGATGTGCCCTCACTACCATCTTGAATG-3´ followed by ligation into HindIII-EcoRV-digested *p3X-FLAG-CMV-13* (Sigma-Aldrich) using the InFusion-Advantage PCR kit (Clontech Laboratories). The integrity of the resulting construct was confirmed by DNA sequencing. The cells were transfected with the indicated plasmids using X-tremeGENE 9 DNA transfection reagent (Roche Diagnostics) according to the manufacturer’s instructions. The cells were harvested 48 hours post transfection, and lysed as described above.

### Statistical Analyses

Statistical analyses were performed to assess the differences between the *Lrig2* genotypes (+/+, +/-, and -/-) using SPSS 14.0 software (IBM, Armonk, NY, USA). The differences in *Lrig* expression in brain were analyzed using the Kruskal-Wallis test. The differences in organ weights were analyzed using independent samples Student’s t-test. Survival was estimated using Kaplan-Meier plots, and the differences between curves were analyzed using the log-rank test. Differences in body weight, *Fos* and *Egr2* expression, and levels of total and phosphorylated proteins were analyzed using the Student’s paired t-test. The relationship between tumor incidence and grade and the mouse genotype was analyzed using the linear-by-linear association chi-squared test. The significance of the association between tumor incidence and mouse genotype was analyzed using Fisher’s exact test. The significance level was set to p<0.05 for all analyses except for the analysis of relative organ weights, where, because of the large number of tests (51 pairwise tests), the significance level was set to p<0.01.

## Results

### The generation of *Lrig2*-Deficient Mice

*Lrig2*-deficient mice were generated by deletion of *Lrig2* exon 12 (referred to as *Lrig2E12*). Exon 12 precedes the region encoding the transmembrane region (Figure 1C), and its ablation results in a frame shift that introduces multiple stop codons. The genomic deletion of *Lrig2E12* was confirmed through Southern blot analysis (Figure 1B) and cDNA sequencing. Western blotting with an antiserum against the cytosolic tail of Lrig2 showed down-regulation of Lrig2 in MEFs from *Lrig2E12+/-* mice and an apparent lack of Lrig2 in MEFs from *Lrig2E12-/-* mice (Figure 1D). The *Lrig* transcript levels in brains of mice of the different *Lrig2* genotypes were analyzed using quantitative real-time RT-PCR (Figure 1E). This analysis showed a significant down-regulation of *Lrig2E17-18*-containing transcripts in both *Lrig2E12+/-* and *Lrig2E12-/-* mice, and a complete lack of *Lrig2E12*-containing transcripts in the *Lrig2E12-/-* mice. There was no significant difference in expression of *Lrig1* or *Lrig3* between mice of the different *Lrig2* genotypes.

### *Lrig2*-Deficient Mice are Born at Mendelian Frequencies but Have Increased Spontaneous Mortality

The current experiments were performed on *Lrig2* genetically modified mice bred onto a C57BL/6 background for at least five generations. When the *Lrig2E12* heterozygotes were mated, their offspring were born at Mendelian frequencies and were equally distributed between males and females with the following genotypic frequencies for males and females, respectively: *Lrig2E12+/+*, 0.28 and 0.22; *Lrig2E12+/-*, 0.50 and 0.53; *Lrig2E12-/-*, 0.22 and 0.25.

**Figure 2:**
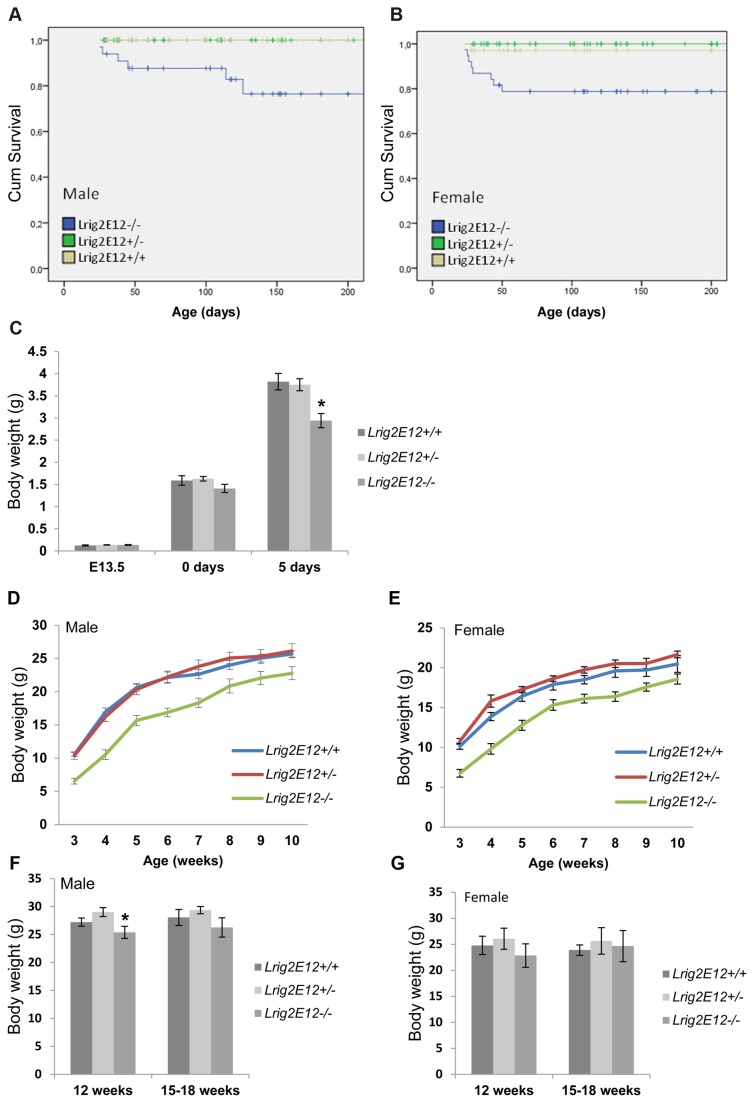
*Lrig2-*deficient mice have increased spontaneous mortality and show transiently reduced body weight compared with wild-type and heterozygous littermates. Kaplan-Meier curves for the survival of male (**A**) and female (**B**) C57BL/6 mice of the different *Lrig2* genotypes based on 153 male and159 female mice. (**C**) There were no significant differences in embryonic weight at E13.5 or at birth, but by 5 days of age, the Lrig2E12-/- mice were significantly lighter than the *Lrig2E12+/+* and *Lrig2E12+/-* mice (* p<0.05). Male (**D**) and female (**E**) *Lrig2E12-/*- mice had a lower body weight than the *Lrig2E12+/+* and *Lrig2E12+/-* mice from 3 to 10 weeks of age. At 12 weeks of age, the male (**F**) (p = 0.019), but not the female (**G**) (p = 0.051), *Lrig2E12-/*- mice were significantly smaller than *Lrig2E12+/+* and *Lrig2E12+/-* mice, and at 15 weeks or older, all genotypes had a similar body weight. At least eight mice were included per genotype and sex for all ages.

The *Lrig2E12-/-* mice showed increased mortality compared with the Lrig2E12+/+ and *Lrig2E12+/-* mice (Figure 2A-B). By 50 days of age, 20% of the female and 12% of the male *Lrig2E12-/-* mice had either died or been euthanized due to disease. At approximately 130 days of age, an additional 12% of the male Lrig2E12-/- mice had either died or been euthanized due to disease. None of the *Lrig2E12+/+* or *Lrig2E12+/-* male mice, and only one of the female *Lrig2E12+/+* mice, had died by the end of the experiment at 200 days of age. As the young *Lrig2E12-/-* mice were smaller than their siblings (see below), it was possible that they were not able to compete successfully with their littermates for nourishment or had problems reaching or chewing solid feed pellets. To prevent malnutrition due to these causes, all of the litters with small pups were given access to feed pellets softened in water and presented on Petri dishes on the floor of the cage. Despite these precautions, mortality remained essentially unchanged. Most of the sick mice died with no prior symptoms of disease. Mice that showed general symptoms of illness (e.g., lethargy, hackled fur, and a crouched body position) were euthanized. Dead or euthanized mice were generally emaciated or in very poor condition but had no other macroscopic signs of disease. Five of the mice (four males and one female) that were euthanized due to illness were submitted for necropsy. These mice were 25 days old, on average, and their average body weight was 5 g (all five mice were emaciated). In one male, histopathological examination showed a moderate purulent cystitis and pyelonephritis. In another male, there was a moderate accumulation of degenerative neutrophils in the lumen of the accessory genital glands, but little or no evidence of epithelial damage. There were no significant histological lesions found in the other tissues investigated from these two mice, and there were no significant findings in the other three mice evaluated.

### *Lrig2*-Deficient Mice Show No Macroscopic Anatomical Defects

The anatomy of apparently healthy 12-week-old mice of the different *Lrig2* genotypes was investigated by visual inspection, and selected organs were weighed. Macroscopically, there were no aberrations or abnormalities observed in any of these mice. There were also no significant changes in the relative organ weights (Table 1; significance level, p<0.01). However, *Lrig2E12-/-* female mice showed a tendency to have larger brains than *Lrig2E12+/+* or *Lrig2E12+/-* female mice (p=0.024 and p=0.049, respectively). There were no detectable histological differences between the *Lrig2E12+/+* and *Lrig2E12-/-* brains (data not shown). In addition, Luxol blue staining did not reveal any difference in the apparent myelin patterns between the brains of the different *Lrig2* genotypes (data not shown).

**Table 1:** Organ weights expressed as % of body weight (standard error of the mean) for 12-week-old, apparently healthy, C57BL/6 mice, with the *Lrig2E12* genotype as indicated (+/+, +/- or -/-).

	Male			Female		
	+/+	+/-	-/-	+/+	+/-	-/-
	(n=4)	(n=11)	(n=9)	(n=9)	(n=12)	(n=10)
Brain	1.65 (0.037)	1.54 (0.044)	1.65 (0.063)	1.69 (0.153)	1.89 (0.073)	2.07 (0.081)
Heart	0.51 (0.024)	0.53 (0.012)	0.52 (0.027)	0.69 (0.190)	0.54 (0.021)	0.53 (0.030)
Lungs	0.53 (0.029)	0.59 (0.064)	0.54 (0.017)	0.59 (0.032)	0.58 (0.023)	0.61 (0.031)
Liver	3.01 (0.896)	3.78 (0.335)	3.91 (0.310)	4.53 (0.337)	4.38 (0.290)	4.50 (0.290)
Spleen	0.30 (0.016)	0.29 (0.015)	0.28 (0.015)	0.34 (0.011)	0.40 (0.030)	0.40 (0.045)
Kidneys	1.34 (0.050)	1.53 (0.038)	1.42 (0.050)	1.14 (0.064)	1.23 (0.028)	1.23 (0.050)
Ovaries	n.a.	n.a.	n.a.	0.32 (0.091)	0.41 (0.079)	0.48 (0.046)
Ventral prostate	0.03 (0.005)	0.03 (0.002)	0.03 (0.002)	n.a.	n.a.	n.a.
Dorsolateral prostate	0.04 (0.006)	0.04 (0.003)	0.04 (0.006)	n.a.	n.a.	n.a.
Seminal vesicles	1.08 (0.072)	0.87 (0.096)	0.99 (0.043)	n.a.	n.a.	n.a.
Testes	0.96 (0.028)	0.95 (0.033)	0.96 (0.036)	n.a.	n.a.	n.a.

n.a., not applicable

### *Lrig2*-Deficient Mice Have Reduced Body Weight Compared to Heterozygous and Wild-Type Mice from 5 Days until 12 to 15 Weeks of Age

The embryo weights at E13.5 and at birth (day 0) did not differ significantly between *Lrig2* genotypes (Figure 2C). At 5 days of age, however, *Lrig2E12-/-* pups were significantly smaller than *Lrig2E12+/+* and *Lrig2E12+/-* pups. This weight difference at 5 days of age was verified in mice with a mixed genetic background (C57BL/6; FVB/N; p<0.05; data not shown). The weight difference remained significant until 12 weeks of age for female mice and until 15 weeks of age for male mice (Figure 2D–G).

### *Lrig2*-Deficient Mice are Protected Against *PDGFB*-Induced Glioma

**Figure 3:**
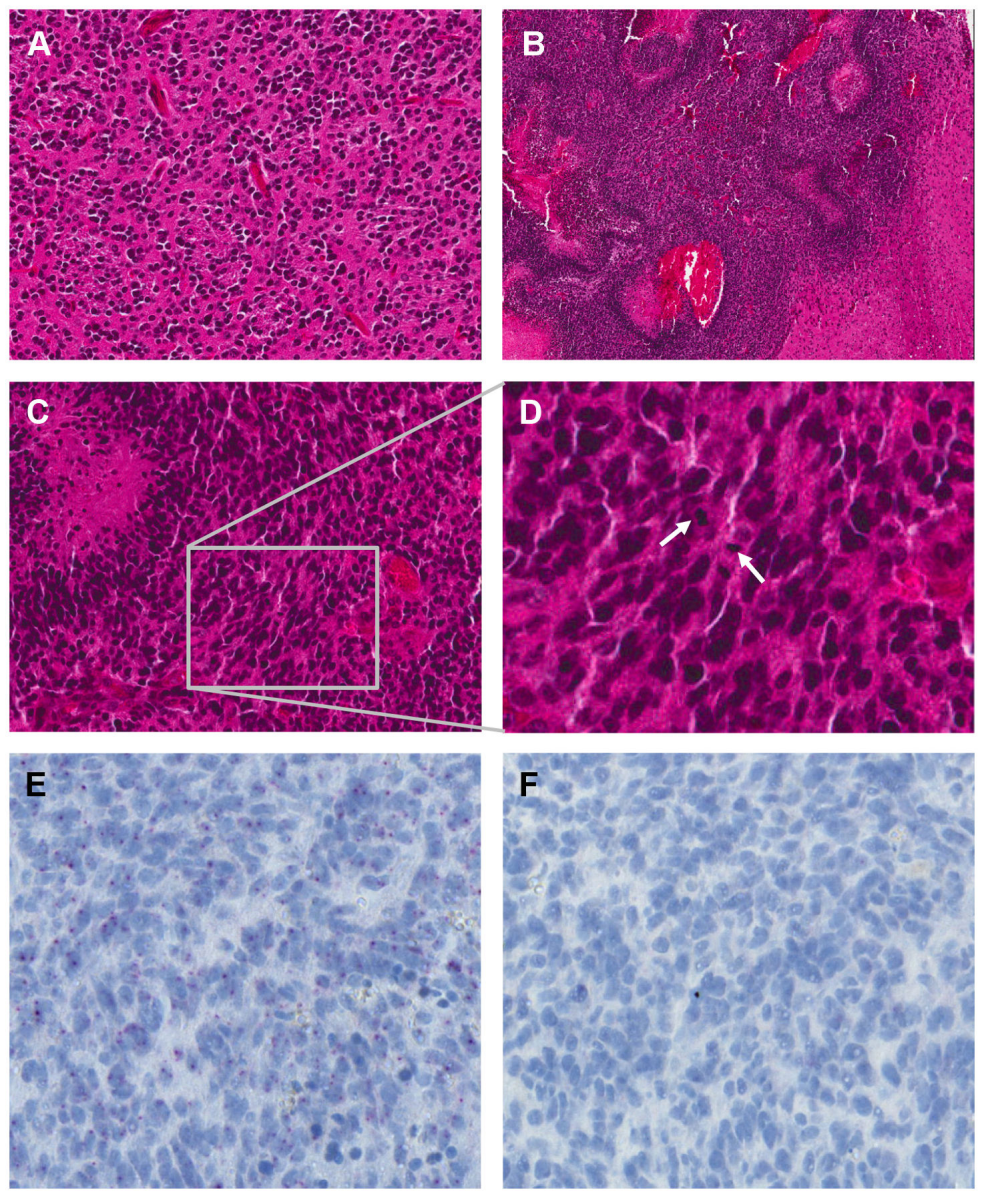
Histological and *in situ* hybridization analyses of *PDGFB*-induced mouse brain tumors. Newborn mice were transduced intracranially with PDGFB-encoding RCAS retroviruses. At 12 weeks of age, the mice were sacrificed, and the histological sections were prepared. (**A**) A hematoxylin-eosin-stained section showing a tumor resembling human oligodendroglioma, WHO grade II/III (low grade). The tumor tissue showed moderate cellularity and was composed of monomorphic cells with uniform, round nuclei. Original magnification 200X. (**B**) A hematoxylin-eosin-stained section showing a tumor resembling human glioblastoma, WHO grade IV (high grade), with pseudopalisading necroses. Original magnification 100X. (**C**) A hematoxylin-eosin stained section showing a high grade glioma with pseudopalisading necroses (arrow) and frequent mitoses. Original magnification 200X. (**D**) Magnification of C, mitoses are shown with arrows. (**E**) *In situ* hybridization analysis of *Lrig2* in high grade glioma. The *in situ* hybridization signals are shown as red dots. Original magnification 400X. (**F**) *In situ* hybridization analysis of the same tumor as in E, using a negative control probe, showing low non-specific background staining.

To evaluate the possible role of *Lrig2* in oligodendroglioma genesis, *Ntv-a* transgenic mice with different *Lrig2* alleles were transduced by intracranial injection with PDGFB-encoding RCAS virus-producing DF-1 cells. This treatment has previously been shown to result in a high frequency of gliomas, predominantly of oligodendroglial histology [5,35]. In some mice, this experimental protocol induced hydrocephalus within three weeks of injection. These mice were euthanized and excluded from the study. The induced tumors were of lower grade (WHO grade II or III), with similarities to human oligodendroglioma, or of higher grade (WHO grade IV), with similarities to human glioblastoma with pseudopalisading necroses and microvascular proliferation (Figure 3A–D). In the tumors of *Lrig2E12+/+* mice, *Lrig2* was expressed, as shown by *in situ* hybridization (Figure 3E-F). Of the mice analyzed, all *Lrig2E12+/+* mice had developed brain tumors at 12 weeks of age (n=22; Table 2). Of these, 82% (18/22) showed low grade lesions, and 18% (4/22) had high grade tumors. In contrast, of the *Lrig2E12-/-* mice (n=13), only 77% (10/13) had developed brain tumors at 12 weeks of age, and none of these tumors were of high grade. Among the *Lrig2E12+/-* mice (n=12), all mice developed gliomas, of which, 92% (11/12) were of low grade and 8% (1/12) were of high grade. The incidence and grade of the tumors was significantly dependent on the *Lrig2* genotype (Table 2, p=0.006, using the linear-by-linear association chi-squared test). Furthermore, the tumor incidence was significantly lower among the *Lrig2E12-/-* mice than among *Lrig2E12+* mice (p=0.018, using Fisher’s exact test). These results show that *Lrig2* promoted the genesis or growth, and malignancy of *PDGFB*-induced glioma.

**Table 2:** Incidence of *PDGFB*-induced glioma for the respective histological grade.

*Lrig2* genotype	No tumor	Low grade tumor	High grade tumor
+/+	0	18/22 (82%)	4/22 (18%)
+/-	0	11/12 (92%)	1/12 (8%)
-/-	3/13 (23%)	10/13 (77%)	0

The tumor incidence and grade was dependent on the *Lrig2* genotype (p=0.006, using linear-by-linear association chi-squared test).

### Lrig2 is Not Required for Retroviral Gene Transduction of Tva-Expressing Neural Cells

**Figure 4:**
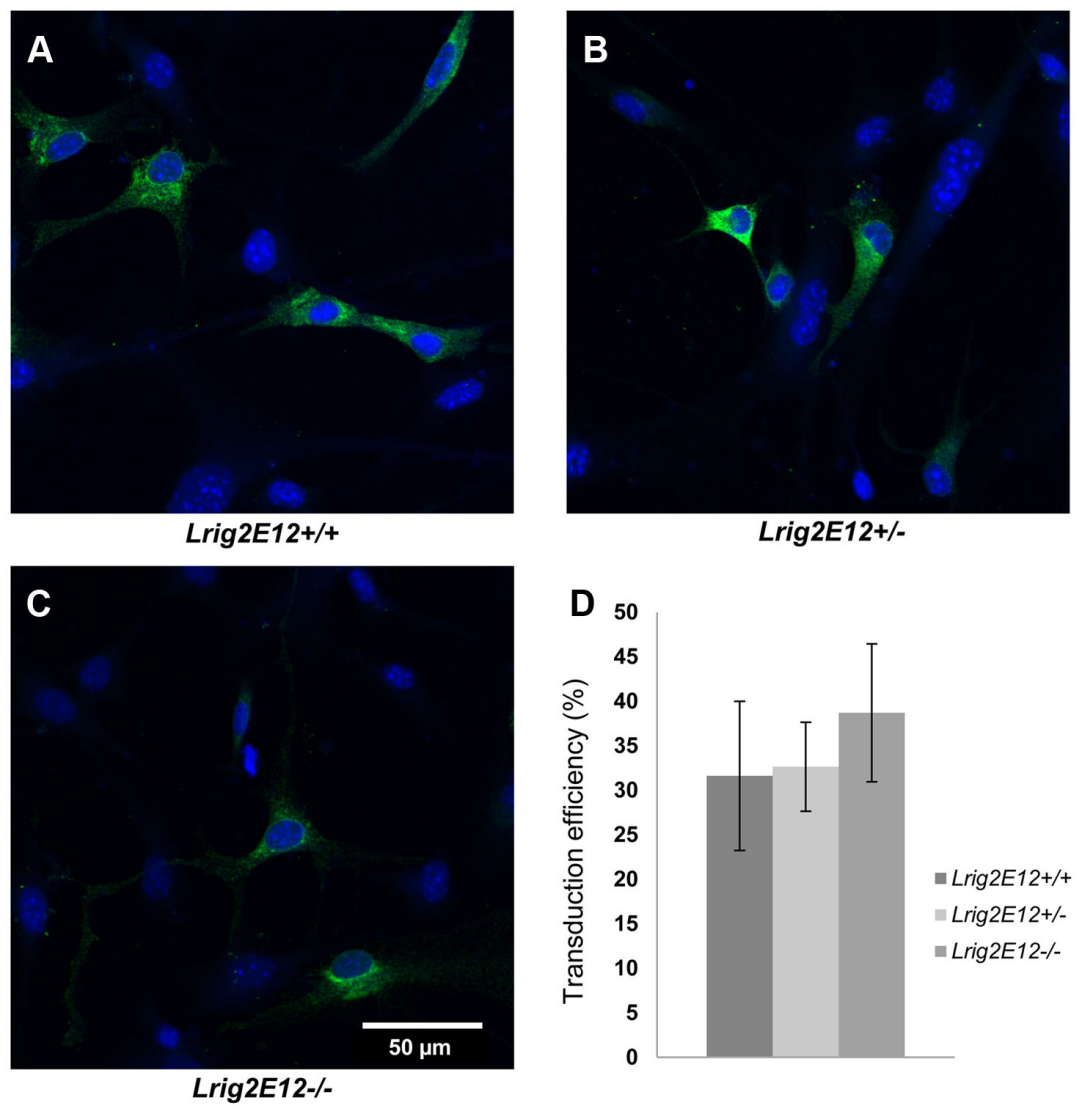
The transduction efficiency of RCAS-PDGFB-HA in *Ntv-a* cells of different *Lrig2* genotypes. Neural cells from brains of newborn *Ntv-a* transgenic mice were transduced with RCAS-PDGFB-HA *in vitro*. After 5 days of infection, cells were stained with fluorescent anti-HA antibodies (green) and analyzed using fluorescence microscopy. Cell nuclei were counter-stained with DAPI (blue). (**A**) Wild-type (*Lrig2E12+/+*) cells. (**B**) Heterozygous (*Lrig2E12+/-*) cells. (**C**) *Lrig2*-defecient (*Lrig2E12-/-*) cells. (**D**) Quantifications of transduction efficiencies. Shown are the means from three independent experiments, including wild-type (n=7), heterozygous (n=12), and *Lrig2*-deficient (n=7) cell lines from three different litters, with standard deviations indicated by error bars. There were no apparent differences in the transduction efficiency between cells of different *Lrig2* genotypes.

To analyze whether the reduced incidence and malignancy of tumors in *Lrig2E12-/-* mice were due to a lower gene transduction efficiency of *Lrig2E12-/-* cells, neural cells from newborn *Ntv-a* transgenic mice of the different *Lrig2* genotypes were transduced with the PDGFB-encoding RCAS viruses *in vitro* (Figure 4). There were no significant differences in transduction efficiency between *Lrig2E12+/+*, *Lrig2E12+/-*, or *Lrig2E12-/-* cells (Figure 4D).

### Lrig2 Does Not Regulate Pdgfr Levels or Primary Cilia Formation

**Figure 5:**
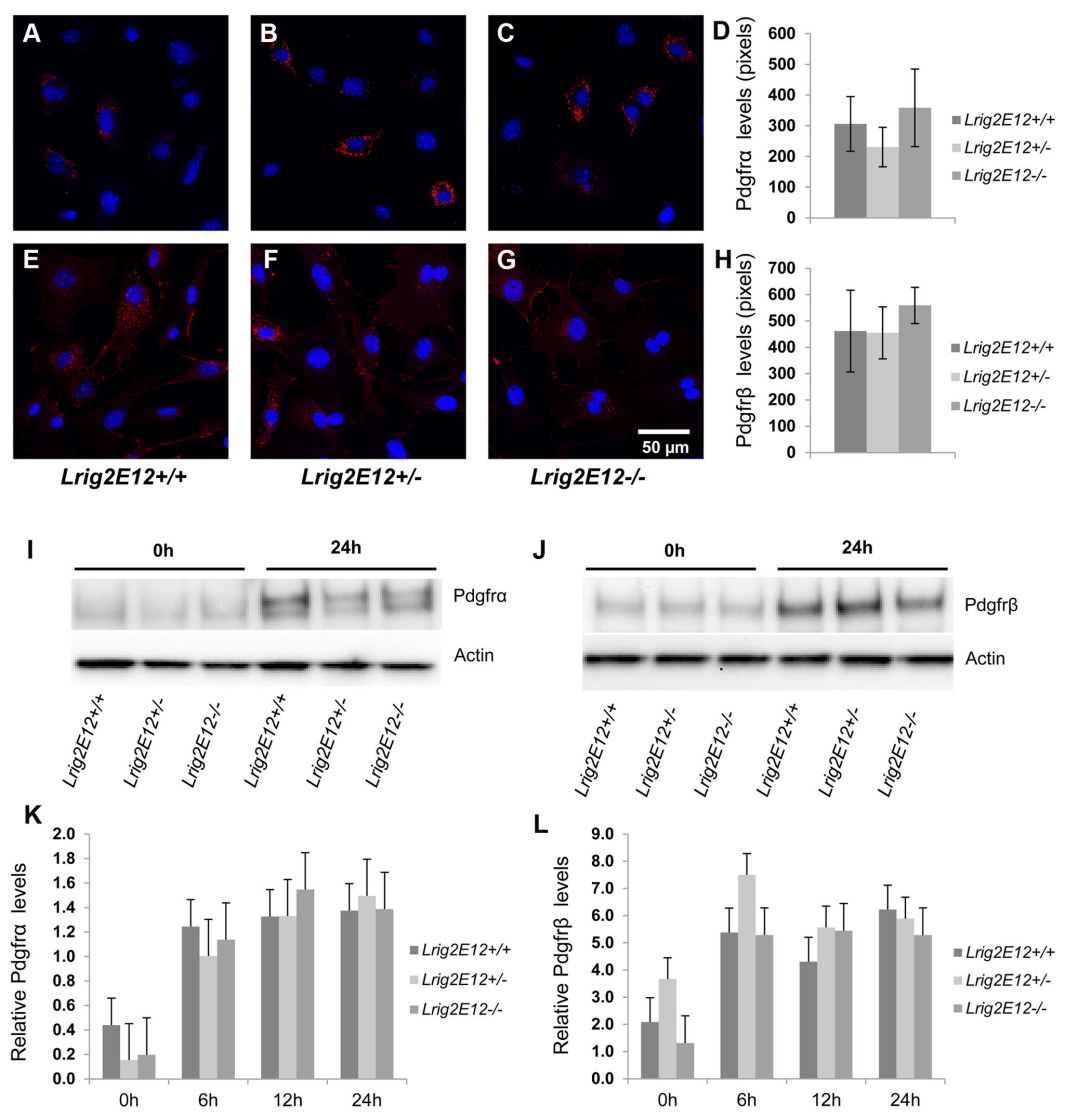
Pdgfr levels in serum-starved primary MEFs. Wild-type (*Lrig2E12+/+*), heterozygous (*Lrig2E12+/-*), or *Lrig2*-deficient (*Lrig2E12-/-*) MEF cell lines were serum-starved for the indicated times followed by analysis of Pdgfr levels using immunofluorescence microscopy (**A**–**H**) or immunoblotting (**I**–**L**). (**A**–**H**) Cells were serum starved for 24 hours followed by analysis of Pdgfrα (**A**–**D**) or Pdgfrβ (**E**–**H**) levels using immunofluorescence microscopy. Pdgfr immunoreactivity was visualized with Alexa-conjugated secondary antibodies (red), and the nuclei were counter-stained with DAPI (blue). (**D**, **H**) Quantifications of the Pdgfrα and Pdgfrβ immunofluorescence levels, respectively. Shown are the means from three independent experiments, including wild-type (n=8), heterozygous (n=9), and *Lrig2*-deficient (n=6) cell lines from three different litters, with standard deviations indicated by error bars. There were no differences observed in Pdgfrα or Pdgfrβ protein levels, as determined by quantitative immunofluorescence microscopy, between cells of different *Lrig2* genotypes. (**I**–**L**) Cells were serum starved for 0, 6, 12 or 24 hours followed by cell lysis and Western blot analyses with antibodies against Pdgfrα, Pdgfrβ, or, as a loading control, actin. Representative immunoblots of Pdgfrα (**I**) and Pdgfrβ (**J**) in wild-type (*Lrig2E12+/+*), heterozygous (*Lrig2E12+/-*), and *Lrig2*-deficient (*Lrig2E12-/-*) cells after 0 or 24 hours of serum starvation. (**K**–**L**) Quantifications of Pdgfrα (**K**) and Pdgfrβ (**L**) immunoblots in wild-type, heterozygotes and *Lrig2*-deficient cells after 0, 6, 12 and 24 h starvation. Shown are the means from three independent experiments including wild-type (n=8), heterozygous (n=9), and *Lrig2*-deficient (n=6) cell lines from three different litters, with standard deviations indicated by error bars. There were no differences observed in Pdgfrα or Pdgfrβ protein levels as determined by immunoblotting, between cells of different *Lrig2* genotypes.

**Figure 6:**
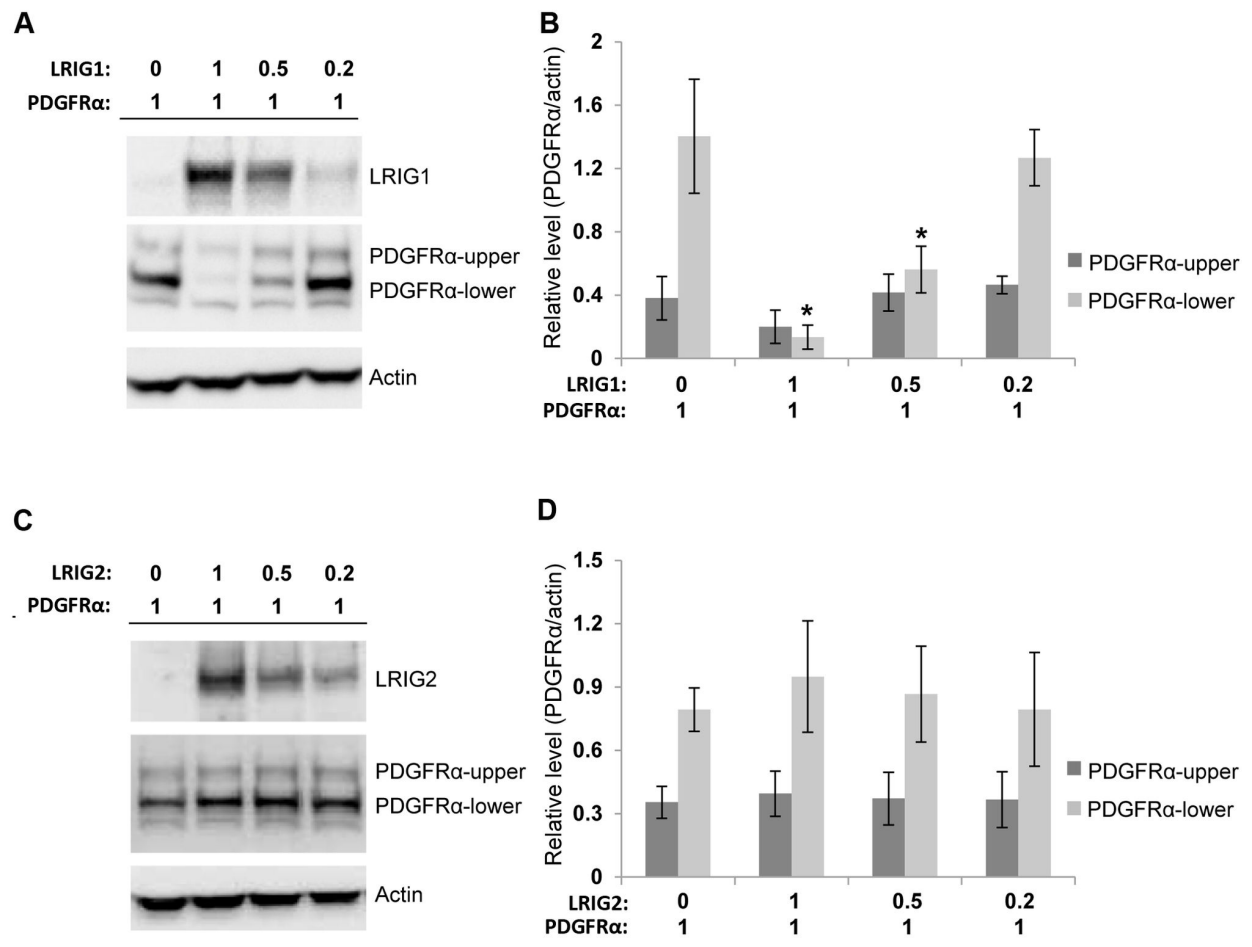
Expression level analyses of co-transfected LRIG1, LRIG2, and PDGFRα. HEK-293T cells were co-transfected with expression vectors encoding myc-LRIG1 or FLAG-LRIG2 and PDGFRα. The numbers indicate amount of plasmid (μg) used in the respective transfection. Empty *pcDNA 3.1* and *p3XFLAG-CMV-13*, respectively, were used to bring the total amount of plasmid DNA to the same amount (2 µg) in each transfection. Cell extracts were analyzed by Western blotting with antibodies against LRIG1, PDGFRα, FLAG (recognizing FLAG-LRIG2), or, as a loading control, actin. (**A**, **C**) Representative Western blots. Two specific PDGFRα bands were observed, here called PDGFRα-upper and PDGFRα-lower, respectively. (**B**, **D**) Quantification of PDGFRα immunoblots. Shown are the means of four independent co-transfection experiments, with error bars indicating the standard deviations. Significant differences compared with the empty vector control are indicated with asterisks (*p<0.05).

**Figure 7:**
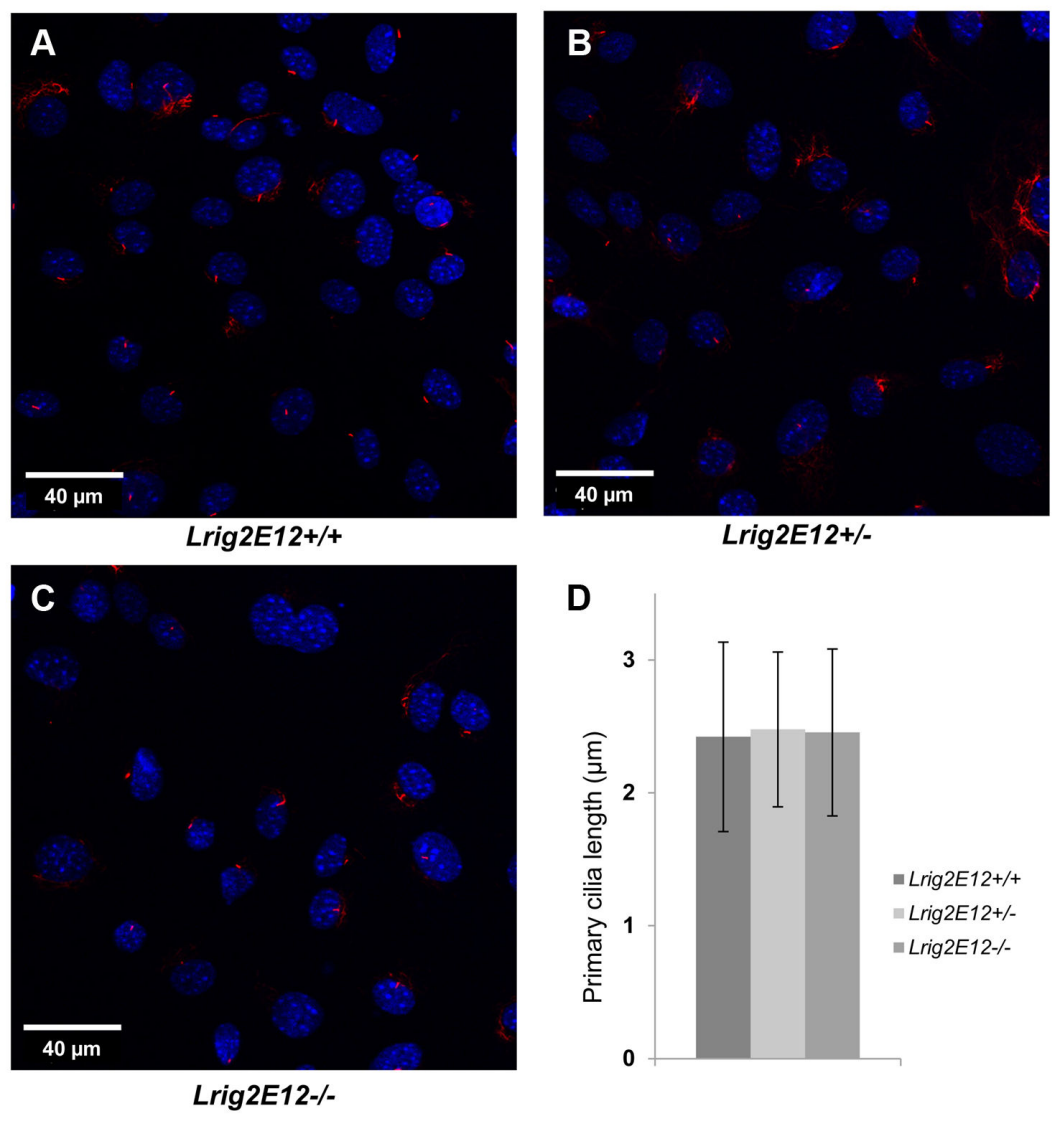
The length of the primary cilia in growth-arrested primary MEFs. Wild-type, heterozygous, and *Lrig2*-deficient cells were serum starved for 24 hours followed by staining of primary cilia with antibodies against acetylated tubulin (red). The cell nuclei were counter-stained with DAPI (blue). Representative confocal immunofluorescence micrographs of: (**A**) wild-type (*Lrig2E12+/+*) cells, (**B**) heterozygous (*Lrig2E12+/-*) cells, and (**C**) *Lrig2*-defecient (*Lrig2E12-/-*) cells. (**D**) Quantification of primary cilia length. Shown are the means from three independent experiments including wild-type (n=8), heterozygous (n=9), and *Lrig2*-deficient (n=6) cell lines from three different litters, with standard deviation indicated by error bars. There were no differences observed in the abundance (data not shown) or the length of primary cilia in cells of different *Lrig2* genotypes.

Pdgfr levels were analyzed in the MEFs of the different *Lrig2* genotypes under standard cell culture conditions using immunofluorescence microscopy (Figure 5A–H). These analyses revealed no difference in Pdgfrα or Pdgfrβ levels between *Lrig2E12+/+*, *Lrig2E12+/-*, or *Lrig2E12-/-* MEFs. Similarly, immunoblot analyses of cell lysates from serum-starved MEFs showed no difference in Pdgfrα or Pdgfrβ levels between *Lrig2E12+/+*, *Lrig2E12+/-*, or *Lrig2E12-/-* cells (Figure 5I–L). To further investigate whether LRIG2 could regulate PDGFR levels, human HEK-293T cells were co-transfected with expression vectors encoding LRIG1 or LRIG2 and PDGFRα. LRIG1 overexpression downregulated the protein expression levels of co-transfected PDGFRα as shown by immunoblot analysis of cell lysates (Figure 6A,B). LRIG2 overexpression, in contrast, did not affect the protein expression levels of co-transfected PDGFRα (Figure 6C,D). Because primary cilia have been shown to be important for signaling by Pdgfrα [36], we analyzed the formation of primary cilia in *Lrig2E12+/+*, *Lrig2E12+/-*, and *Lrig2E12-/-* MEFs. There was no difference in the abundance or length of starvation-induced primary cilia between the cells of different *Lrig2* genotypes (Figure 7).

### The Kinetics of Immediate-Early Gene-Induction are Altered in *Lrig2*-Deficient Cells

**Figure 8:**
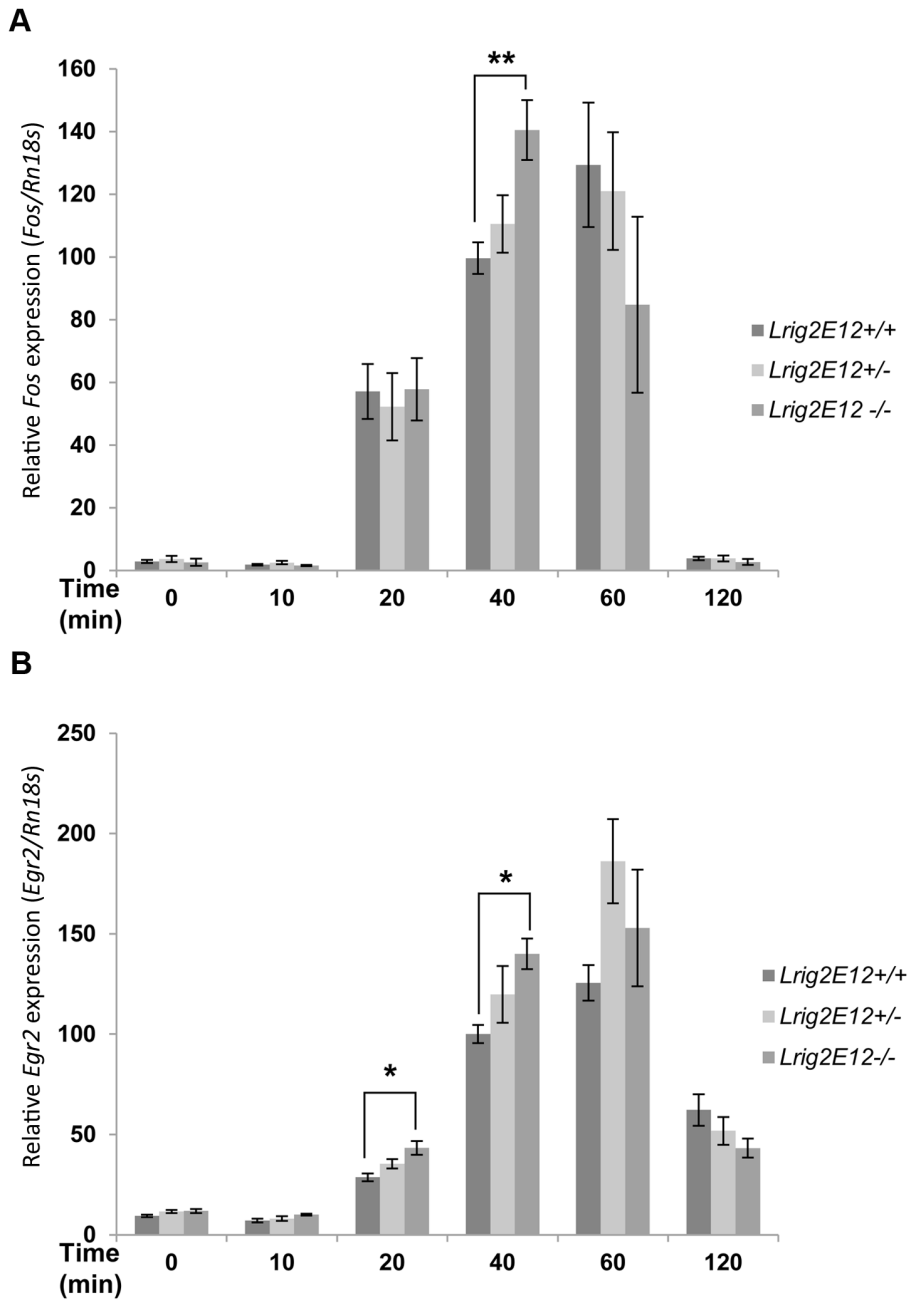
The effect of *Lrig2* on the induction of immediate-early gene expression. Wild-type (*Lrig2E12+/+*), heterozygous (*Lrig2E12+/-*), or *Lrig2*-deficient (*Lrig2E12-/-*) MEFs were serum starved for 24 hours followed by stimulation with PDGF-BB for 0, 10, 20, 40, 60, or 120 minutes. Thereafter, total RNA was prepared, and gene expression was quantified using real-time RT-PCR. Samples were run in triplicate, and the specific mRNA levels were normalized to respective *Rn18s* levels. Shown are the specific mRNA/*Rn18s* ratios on arbitrary scales. (**A**) Kinetics of relative *Fos* expression in cells stimulated with 50 ng/ml PDGF-BB. Shown are the means from three independent experiments, including wild-type (n=8), heterozygous (n=9), and *Lrig2*-deficient (n=6) cell lines from three different litters, with standard error of the means indicated by error bars. (**B**) Kinetics of relative *Egr2* expression levels in cells stimulated with 10 ng/ml PDGF-BB. Shown are the means from two independent experiments, including wild-type (n=6), heterozygous (n=6), and *Lrig2*-deficient (n=4) cell lines from two different litters, with standard error of the means indicated by error bars. Compared with wild-type cells, the *Lrig2*-deficient cells showed altered and faster kinetics of induction of both *Fos* and *Egr2* expression in response to PDGF-BB stimulation. Significant differences compared with the wild-type cell lines are indicated with asterisks (*p<0.05 and **p<0.01).

To investigate whether Lrig2 regulates PDGF signaling, we analyzed MEF cell lines from *Lrig2E12+/+*, *Lrig2E12+/-*, and *Lrig2E12-/-* embryos. When these cells were stimulated with PDGF-BB, expression levels of the immediate-early genes *Fos* (also called *c-Fos*) and *Egr2* were induced (Figure 8). Intriguingly, the *Lrig2E12-/-* cells showed an altered and more rapid kinetic profile of *Fos* and *Egr2* induction than the *Lrig2E12+/+* cells. The *Lrig2E12-/-* cells displayed 41% higher *Fos* levels at 40 minutes (p<0.01) and 35% lower *Fos* levels at 60 minutes (not significant) than the *Lrig2E12+/+* cells after stimulation with 50 ng/ml PDGF-BB (Figure 8A). The *Lrig2E12+/-* cells showed *Fos* levels that were intermediate to the levels of *Lrig2E12+/+* and *Lrig2E12-/-* cells at these time points. Similar results were obtained when using a lower concentration of PDGF-BB (10 ng/ml) for *Fos* (p<0.05 at 40 minutes, data not shown) and for *Egr2* (Figure 8B). *Lrig2E12-/-* cells stimulated with 10 ng/ml PDGF-BB showed 51% higher levels of *Egr2* at 20 minutes (p=0.03), 40% higher levels at 40 minutes (p<0.01), and 31% lower levels at 120 minutes (not significant) than *Lrig2E12+/+* cells.

### Lrig2 Appears to Not Regulate Pdgfr, Akt, or Erk1/2 Phosphorylation Levels

**Figure 9:**
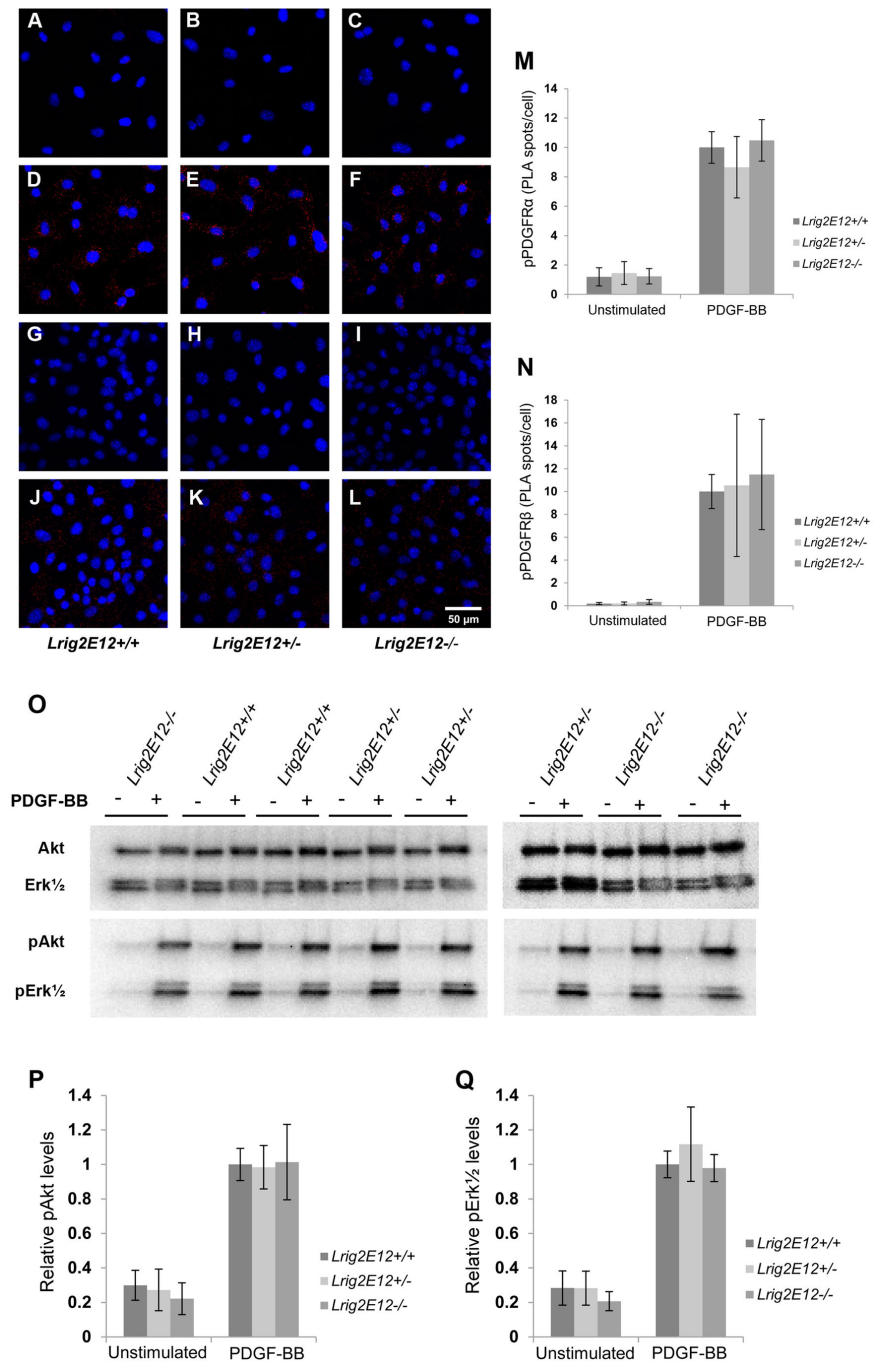
PDGF induced phosphorylation events in cells of different *Lrig2* genotypes. Wild-type, heterozygous, or *Lrig2*-deficient MEFs were serum starved for 24 hours followed by stimulation with 50 ng/ml PDGF-BB for different times. (**A**–**N**) Cells were untreated or stimulated with 50 ng/ml PDGF-BB for 10 minutes followed by cell fixation and analysis of the phosphorylation status of respective Pdgfr by *in situ* proximity ligation assay (PLA). Phosphorylated Pdgfr was visualized using fluorescence (red spots). Cell nuclei were counter-stained with DAPI (blue). (**A**–**F**) Representative PLA images of phosphorylated Pdgfrα (red spots) in un-stimulated (**A**–**C**) or PDGF-BB stimulated (**D**–**F**) cells of the indicated *Lrig2* genotypes. (**G**–**L**) Representative PLA images of phosphorylated Pdgfrβ (red spots) in non-stimulated (**G**–**I**) or PDGF-BB-stimulated (**J**–**L**) cells of the indicated *Lrig2* genotypes. (**M**) Quantification of PLA spots for phosphorylated Pdgfrα. Shown are the means from three independent experiments, including wild-type (*Lrig2E12+/+*, n=8), heterozygous (*Lrig2E12+/-*, n=9), and *Lrig2*-deficient (*Lrig2E12-/*-, n=6) cell lines from three different litters, with standard deviations indicated by error bars. (**N**) Quantification of PLA spots for phosphorylated Pdgfrβ. Shown are the means from three independent experiments, including wild-type (*Lrig2E12+/+*, n=8), heterozygous (*Lrig2E12+/-*, n=9), and *Lrig2*-deficient (*Lrig2E12-/*-, n=5) cell lines from three different litters, with standard deviations indicated by error bars. There were no differences observed in the levels of activated Pdgfrα or Pdgfrβ between cells of different genotypes. (**O**–**Q**) Cell lysates from cells that had been untreated or treated with 50 ng/ml PDGF-BB for 15 minutes were analyzed through Western blotting with antibodies against the indicated proteins. (**O**) A representative Western blot is shown of Akt, Erk1/2, phosphorylated Akt (pAkt), and phosphorylated Erk1/2 (pErk1/2) using cell lysates from cells of the indicated *Lrig2* genotypes that had been untreated (-) or treated (+) with PDGF-BB. (**P**) Quantification of pAkt/Akt-ratios for non-stimulated and stimulated cells, respectively. Shown are the means from three independent experiments, including wild-type (n=8), heterozygous (n=9), and *Lrig2*-deficient (n=5) cell lines from three different litters, with standard deviations indicated by error bars. (**Q**) Quantification of pErk1/2/Erk1/2-ratios for non-stimulated and stimulated cells, respectively. Shown are the means and corresponding standard deviations as for **P**.

Pdgfr autophosphorylation was analyzed using PLA before and after stimulation with PDGF-BB (Figure 9A–N). Stimulation of cells with PDGF-BB induced enhanced phosphorylation levels of the Pdgfrs; however, there was no apparent difference in the Pdgfr phosphorylation levels between cells of the different *Lrig2* genotypes. Neither did immunoblot analysis of MEF lysates reveal any differences between the *Lrig2* genotypes in their PDGF-BB induced or un-induced phosphorylation levels of the Pdgfr downstream targets Akt or Erk1/2 (Figure 9O–Q).

## Discussion

This is the first report of an *Lrig2*-deficient mouse strain. Our study revealed a role for *Lrig2* in mouse growth and survival, gliomagenesis, and cell signaling. *Lrig2* was disrupted by ablation of exon 12, which resulted in an altered reading frame, introducing multiple stop codons in the corresponding mRNA. Exon 12 encodes the C-terminal portion of the LRR domain, preceding the immunoglobulin-like, transmembrane, and cytosolic domains. Thus, the *Lrig2E12-/-* mice could not express full-length Lrig2. However, expression of a truncated Lrig2 protein lacking the immunoglobulin-like, transmembrane, and cytosolic domains remained a possibility. Due to a lack of reagents, e.g., suitable antibodies, we were unable to determine whether a truncated Lrig2 protein was expressed in *Lrig2E12-/-* mice. However, we find it unlikely that the phenotype observed was the result of non-physiological functions of a hypothetical and truncated Lrig2 protein. First, the *Lrig2* transcript levels were down-regulated in the *Lrig2E12-/-* mice compared to wild-type mice, making the risk of non-specific and non-physiologic interactions less likely. Second, in a genetic screen in the nematode *Caenorhabditis elegans*, it was found that mutations introducing stop codons after the first Ig domain in the single nematode LRIG gene, *SMA-10*, resulted in similar, or identical, phenotypes as a true null-allele [27]. Thus, and as the observed phenotype could clearly be attributed to the *Lrig2E12* allele, we propose that *Lrig2* plays important roles in the processes leading to the phenotypes observed. Little is known regarding the regulation of *Lrig* expression, and it is not known whether the Lrig proteins have redundant functions. However, our quantifications of the *Lrig* transcript levels in mice of the different *Lrig2* genotypes indicated that neither *Lrig1* nor *Lrig3* was feedback regulated by *Lrig2*; at least, not in the brain.

*Lrig2E12-/-* mice showed increased spontaneous mortality and transiently reduced growth rates. The majority of the deaths occurred during the first week after weaning. By 130 days of age, between 20 and 25% of *Lrig2E12-/-* mice had either died or been euthanized due to the sudden onset of severe, general symptoms of illness. Frequently, these mice were emaciated, but the cause of emaciation or any other illness symptoms could not be determined by routine necropsy. Similarly, no anatomical or histological abnormalities were found in apparently healthy *Lrig2E12-/-* mice. Therefore, the increased spontaneous mortality of *Lrig2E12-/-* mice remains unexplained. Reduced body size is a common and often idiopathic effect of genetic modifications in mice, and there are numerous reports of genetically modified mouse strains with reduced body size [37]. In our *Lrig2E12-/-* mice, however, the reduction in body weight was only transient. The weight differences between *Lrig2E12+/+* and *Lrig2E12-/-* mice were obvious at 5 days of age, and the differences remained until 12 to 15 weeks of age, after which, the differences disappeared. A similar transient reduction in body size or growth retardation is observed in *Pa2g4* (also called *ErbB3-binding protein 1* or *Ebp1*) knockout mice [38] and *Otx1* knockout mice [39]. The *Otx1* knockout mice also show transient hypogonadism, and their transient growth retardation is thought to be secondary to low levels of pituitary hormones. By 4 months of age, *Otx1* knockout mice display restored growth, gonadal function, and pituitary hormone production. In the case of the *Lrig2E12-/-* mice, we are not yet able to explain the mechanisms underlying the transient weight reduction and subsequent recovery; however, this observation may indicate that Lrig2 exerts temporally restricted effects on growth regulation via specific cell types, which was also suggested for ErbB3-binding protein 1 [37].

Intriguingly, *Lrig2E12-/-* mice were protected against *PDGFB*-induced glioma. The current investigation was prompted by our previous finding that LRIG2 expression is associated with poor oligodendroglioma patient survival [31]. In oligodendroglial tumors, dysregulated PDGFR signaling is a common feature. Therefore, we used an animal model of PDGF-induced glioma where PDGFB-encoding RCAS retroviruses are injected intracranially into *Ntv-a* transgenic mice. As previously reported [5], this treatment resulted in brain tumors resembling human oligodendroglioma and glioblastoma. *Lrig2E12+/+* animals developed tumors at a higher frequency and of higher malignancy than *Lrig2E12-/-* mice. This result implies a promoting role of Lrig2 in the genesis and/or progression of oligodendroglioma and in PDGF signaling. A previous report showed that the level of PDGF signaling in the current glioma model determines tumor latency and malignancy [40]. Therefore, we speculate that Lrig2 might have positively regulated PDGFR signaling in the tumors of wild-type mice. To address whether Lrig2 regulates Pdgfr signaling, we analyzed PDGF-BB induced signaling events in a series of primary MEF cell lines that were established from inbred mice of different *Lrig2* genotypes. We chose to study PDGF signaling in these cells because primary MEFs represent a well-characterized and reproducible experimental system for the study of PDGF signaling. These analyses revealed subtle but significant changes in Pdgfr signaling in the *Lrig2E12-/-* cells. The *Lrig2E12-/-* cells showed a more rapid induction of the immediate-early genes *Fos* and *Egr2* in response to PDGF stimulation than *Lrig2E12+/+* cells. The kinetic difference, although subtle, was reproducible and significant, showing that Lrig2 influenced Pdgfr signaling in these cells. Intriguingly, we were unable to demonstrate any effect of Lrig2 on the Pdgfr protein levels or phosphorylation events of Pdgfr, Akt, or Erk. Similarly, we failed to detect any difference in Pdgfrα or Pdgfrβ levels in the tumors of different *Lrig2* genotypes by immunohistochemistry (data not shown). This apparent paradox of an observable effect on immediate-early gene induction but not on Pdgfr, Akt, or Erk phosphorylation levels could be the result of different mechanisms. For example, the regulatory function of Lrig2 could be downstream of the Pdgfrs, Akt, and Erk, or at parallel pathways that were not analyzed in the present study. Another possibility is that Lrig2 regulates the cytoplasmic-nuclear translocation of transcription factors or other regulatory molecules. It is also possible that our assays used to monitor protein phosphorylation levels were not sensitive enough to detect small changes in phosphorylation levels that might have been translated into detectable differences in gene induction. Thus, Lrig2 clearly regulated PDGF-induced immediate-early gene induction; however, the molecular mechanism for this regulation remains to be clarified. Because the gliomas in our animal model were induced by *PDGFB*, we find it likely that the tumor promoting effect of Lrig2 was mediated by regulation of PDGF signaling. However, other tumor-promoting effects of Lrig2 cannot be excluded, at present. Likewise, the current data cannot discriminate between cancer cell autonomous and non-autonomous effects of Lrig2. In this regard, it is noteworthy that the survival benefit of low LRIG2 expression in human oligodendroglioma patients was due to LRIG2 expression in the cancer cells only [31]. This observation may suggest that the tumor promoting effect of LRIG2 is indeed cancer cell autonomous. In any case, the results herein presented, showing that Lrig2 promoted *PDGFB*-induced glioma in mice, are consistent with the previous finding that LRIG2 expression predicts poor survival in human oligodendroglioma patients [31]. Taken together, these two observations suggest that LRIG2 is an important regulator of oligodendroglioma initiation or progression.

## Conclusions

The phenotype of *Lrig2E12-/-* mice was distinct from that of the previously described *Lrig1*- and *Lrig3*-mutant mice, and included increased spontaneous mortality, transiently reduced growth rate, and protection against *PDGFB*-induced glioma. In addition, *Lrig2E12-/-* MEFs showed altered kinetics of immediate-early gene induction following stimulation with PDGF-BB. These results show that Lrig2 promotes glioma and regulates growth factor signaling in a manner distinct from that of Lrig1.
